# Phylogenetic relationships and pathogenicity variation of two Newcastle disease viruses isolated from domestic ducks in Southern China

**DOI:** 10.1186/1743-422X-11-147

**Published:** 2014-08-12

**Authors:** Yinfeng Kang, Yanling Li, Runyu Yuan, Xianwei Li, Minhua Sun, Zhaoxiong Wang, Minsha Feng, Peirong Jiao, Tao Ren

**Affiliations:** National and Regional Joint Engineering Laboratory for Medicament of Zoonosis Prevention and Control, Guangzhou, China; Key Laboratory of Animal Vaccine Development, Ministry of Agriculture, Guangzhou, China; Key Laboratory of Zoonosis Prevention and Control of Guangdong, Guangzhou, China; College of Veterinary Medicine, South China Agricultural University, 483 Wushan Road, Tianhe District, Guangzhou, 510642 P.R. China

**Keywords:** Newcastle disease virus, Phylogenetic analysis, Pathogenicity, Transmission

## Abstract

**Background:**

Newcastle disease (ND) is an OIE listed disease caused by virulent avian paramyxovirus type 1 (APMV-1) strains, which is enzootic and causes large economic losses in the poultry sector. Genotype VII and genotype IX NDV viruses were the predominant circulating genotype in China, which may possibly be responsible for disease outbreaks in chicken flocks in recent years. While ducks and geese usually have exhibited inapparent infections.

**Methods:**

In the present study, we investigate the complete genome sequence, the clinicopathological characterization and transmission of two virulent Newcastle disease viruses, SS-10 and NH-10, isolated from domestic ducks in Southern China in 2010.

**Results:**

F, and the complete gene sequences based on phylogenetic analysis demonstrated that SS-10 (genotype VII) and NH-10 (genotype IX) belongs to class II. The deduced amino acid sequence was ^(112)^R-R-Q-K/R-R-F^(117)^ at the fusion protein cleavage site. Animal experiment results showed that the SS-10 virus isolated from ducks was highly pathogenic for chickens and geese, but low pathogenic for ducks. It could be detected from spleen, lung, kidney, trachea, small intestine, bursa of fabricius, thymus, pancreas and cecal tonsils, oropharyngeal and cloacal swabs, and could transmit to the naive contact birds. Moreover, it could transmit to chickens, ducks and geese by naive contact. However, the NH-10 virus isolated from ducks could infect some chickens, ducks and geese, but only caused chickens to die. Additionally, it could transmit to the naive contact chickens, ducks, and geese.

**Conclusion:**

The two NDV isolates exhibited different biological properties with respect to pathogenicity and transmission in chickens, ducks and geese. Therefore, no species-preference exists for chicken, duck or goose viruses and more attention should be paid to the trans-species transmission of VII NDVs between ducks, geese and chickens for the control and eradication of ND.

**Electronic supplementary material:**

The online version of this article (doi:10.1186/1743-422X-11-147) contains supplementary material, which is available to authorized users.

## Background

Newcastle disease (ND) is caused by specified viruses of the avian paramyxovirus type I (APMV-1) serotype of the genus Avulavirus belonging to the family Paramyxoviridae [1]. There are 11 serotypes of avian paramyxoviruses designated APMV-I to APMV-11 and Newcastle disease virus (NDV) has been designated APMV-1. Since its recognition in 1926, ND is regarded as being endemic in many countries. NDV is characterized by pathogenesis in chickens and has been categorized into three main pathotypes, based on the clinical signs seen in infected chickens [2]. Lentogenic isolates are of low virulence and cause mild respiratory or enteric infections. Viruses of intermediate virulence can cause primarily respiratory disease, while viruses of velogenic virulent can cause high mortality. Velogenic forms of NDV are further classified as neurotropic or viscerotropic based on clinical manifestation. OIE defines ND as an infection caused by an APMV-1 virus with an intracerebral pathogenicity index (ICPI) of 0.7 or greater in day-old chickens or by multiple basic amino acids at the F protein cleavage site [3]. Previous studies had compared the precursor peptide F amino acid sequences of NDV strains that varied in their virulence, and showed that virulent viruses had the amino acid sequence ^112^R/K-R-Q-K/R-R^116^ at the C terminus of the F2 protein and phenylalanine at residue 117 at the N terminus of the F1 protein. However, viruses with low levels of virulence had the sequence ^112^ G/E-K/R-Q-G/E-R^116^ at the C terminus of the F2 protein and leucine at residue 117 at the N terminus of the F1protein [4–9].

In China, Newcastle disease was first described in 1946 [10] and, since then, outbreaks have been regularly reported in the rural poultry of Southern China [11–13]. Genotype VII NDV viruses were the predominant circulating genotype in China, which may possibly be responsible for disease outbreaks in chicken flocks in recent years. While the genotype IX viruses, to which the China challenge strain F48E8 used for vaccine evaluation belongs, were found only in China and still induced sporadic infections in certain areas [10]. Additionally, ducks and geese usually have inapparent infections, but some isolates (genotypes VII and VI) have caused outbreaks among geese in China since the 1990s [12, 14]. Clinical cases have been also been described occasionally in ducks.

In the present study, two NDVs isolated from outbreaks in domestic ducks in China during 2010 were genotypically and pathotypically characterized. The pathogenicity and transmission of SS-10 strain and NH-10 strain were evaluated in chickens, ducks and geese, which provided experimental reference data to the integrate control and prevention of ND in waterfowl.

## Results

### Biological characterization assessment of the two isolates

The initial biological characterizations of the two NDV isolates, including the MDT and ICPI, are presented in Table 1. This table also shows the virulence index of these viruses, the MDT, ICPI of isolate SS-10 were 51.6 h and 1.89, while those of NH-10 were 58.8 h and 1.7, which were typical of velogenic NDV strains.

**Table 1:** **Sequential information and pathogenicity of two viruses in this study**

Isolate name	Host	Separatum	Isolate year	EID _50_/0.2 ml	ICPI ^a^	MDT(h) ^b^	Fusion protein cleavage site ^c^
SS-10	Duck	Sanshui	2010	10 ^-9.83^	1.89	51.6	^112^RRQKRF^117^
NH-10	Duck	Nanhai	2010	10^-9.62^	1.7	58.8	^112^RRQRRF^117^

^a^A strain with an ICPI below 0.7 was considered to be lentogenic, while those with an ICPI equal to or greater than 1.60 were considered to be velogenic, and those with ICPI values between 0.7 and 1.60 were considered to be mesogenic. The ICPI of avirulent viruses were near 0.00 [2].

^b^For 9-day-old embryonated chicken eggs, the mean death time (MDT) value (h) of more than 90 was defined as lentogenic; 60–90 as mesogenic, and under 60 as velogenic.

^c^Cleavage site sequences (residues 112 to 117) of the F protein.

### Sequence analysis

The results of virulence tests, as determined by the MDT and ICPI tests, were for the most part, in accordance with those determined by the F0 protein proteolytic cleavage site motifs (residues 112 to 117). Strain SS-10 shared the cleavage site motif ^112^RRQKRF^117^, while strain NH-10 contained the ^112^RRQRRF^117^, which are a molecular characteristic of virulent NDV strains. The nucleotide sequence data were deposited into the GenBank database and the accession numbers were KF219497 (SS-10) and KF219498 (NH-10). The genome of the two isolates (SS-10, NH-10) are 15,192 nt in length, containing a six nucleotides insertion in the 5′-noncoding region (NCR) of the nucleoprotein gene between positions 1738 and 1743 and G + C content of 46.48%, 46.72%, respectively. Additionally, nucleotide sequences of the 3′ leader and 5′ trailer regions of the two NDV isolates which have identical to length, 55 nt or 114 nt, respectively. The two NDV isolates contain mRNA edit site (UUUUUCC↓C) in positions 484 nt of the P gene.

### Phylogenetic analysis

**Figure 1:**
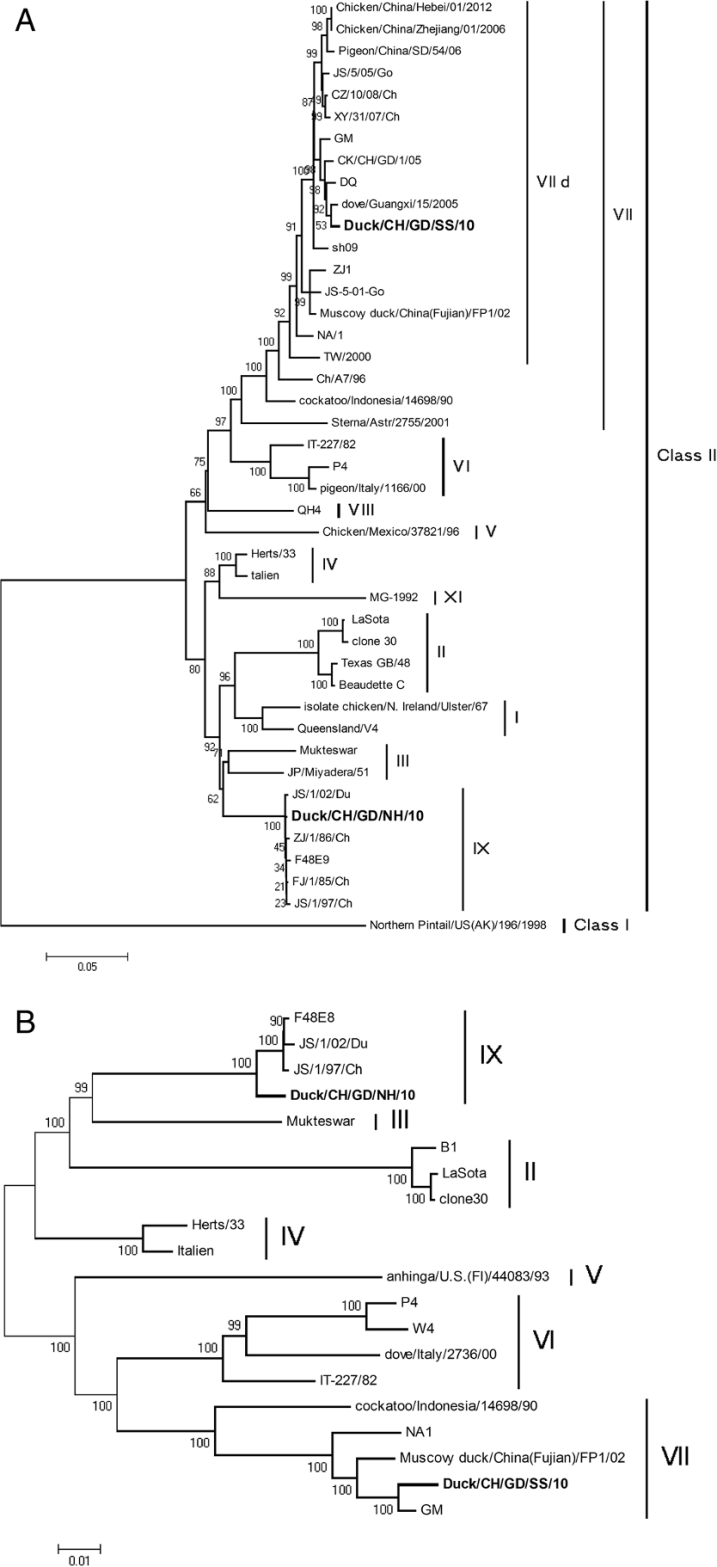
**Phylogenetic relationships of the nucleotide sequences of 62 NDV strains based on the entire ORF (1 to 1,662 nt) of the F gene (A) and the complete gene (B) (nt 1 to 15192).** Sequences previously published in GenBank are listed in Materials and methods and Additional file 1: Table S1. The phylogram was generated by the neighbor-joining method using MEGA 4 software [16].

According to the phylogenetic tree of F gene (nt 1 to 1662) and the complete genome (1 to 15192 nt) [15] which were based on 62 genome-scale that is shown in (Figure 1). The phylogeny showed that SS-10 isolated from Guangdong-Sanshui belonged to genotype VII, and NH-10 isolated from Guangdong-Nanhai belonged to genotype IX. The whole genome sequences of the two isolates and 62 reference strains have high homologies of 83.6% to 99.8%. Strain SS-10 showed greatest nucleotide identities (99.2%) with the velogenic strain GM (Accession number DQ486859). In addition, the isolate was highly similar to ZJ/1, CK/CH/GD/1/05, CZ/10/08/CH, and dove/Guangxi/15/2005, which displayed a nucleotide sequence homology of 96.6 to 98.9%. Strain NH-10 showed higher sequence homology among strains having the same genotype. For example, Strain NH-10 (genotype IX) had nucleotide homologies of 99.8%, 99.7%, 99.7%, 99.6% with JS/1/02/Du, JS/1/97, and ZJ/1/86/CH, respectively. The homologies of nucleotide sequences were relatively low between different genotypes. The F-gene full-length amino acid sequences of the two field isolates were compared with those of reference viruses representing different genotypes. The results showed that SS-10 and NH-10 had sequence homologies of 83.6% and 89.3% at the nucleotide level with vaccine strain Lasota, respectively. No recombination events were observed in the two isolates (SS-10, NH-10) genome (date not shown).

#### Animal experiment

**Pathogenicity and transmission in chickens** In order to investigate the pathogenicity of these three viruses in chickens, we inoculated intranasally with 10^6^ EID_50_ of SS-10 or NH-10 viruses in a volume of 200 μl in SPF chickens, respectively. Then all the chickens were observed for 14 days after infection.

All the chickens of the SS-10 group died within 6 DPI. All the chickens inoculated with NH-10 died within 5 DPI (Table 2). The SS-10 and NH-10 viruses replicated systemically in chickens, which could be detected from all the tested organs on 3 DPI, including the heart, liver, spleen, lung, kidney, brain, trachea, small intestine, bursa of fabricius, thymus, pancreas and cecal tonsils. The SS-10 or NH-10 viruses replicated highly in lung; the mean titers were 8.33 log_10_EID_50_ and 7.5 log_10_EID_50_, respectively (Table 3). In addition, the SS-10 or NH-10 viruses replicated in lymphoid tissues(spleen, bursa of fabricius, thymus and cecal tonsils), whose virus titers were from 6.67 log_10_EID_50_ to 7.5 log_10_EID_50_.

**Table 2:** **Lethality in chicken, ducks and geese of NDV after inoculated intranasally**

Strains	Manifestations of chicken		Manifestations of ducks		Manifestations of geese	
	No. D/S/total ^a^	No. S.C./total ^b^	No. D/S/total	No. S.C./total	No. D/S/total	No. S.C./total
SS-10	3/3/3	1/3	0/1/3	3/3	1/1/3	3/3
NH-10	3/3/3	- ^c^	0/0/3	3/3	0/1/3	3/3

^a^No. D/S/total shows the number of dead (D) and sick (S) as well as the total number of animals from the observation period. The animals that showed disease signs, such as depression and ruffled feathers, but recovered at the end of the observation were counted as sick animals.

^b^No. S.C./total shows the number of animals that seroconverted out of the total number of ducks at the end of the observation period.

^c^All the chickens died at the end of the observation.

**Table 3:** **Replication in chickens of NDV after inoculated intranasally** ^**a**^

Virus replication on 3 DPI (log _10_EID _50_/g) ^b^ in:	Strains	
	SS-10	NH-10
**Heart**	4.56 ± 0.88	4.58 ± 0.58
**Liver**	5.5 ± 0.25	5.42 ± 0.14
**Spleen**	7.42 ± 0.14	7.08 ± 0.29
**Lung**	8.33 ± 0.14	7.5 ± 0.25
**Kidney**	6.17 ± 0.38	5.83 ± 0.63
**Brain**	5.08 ± 0.58	4.5 ± 0.91
**Trachea**	6.08 ± 0.29	5.08 ± 0.76
**Small intestine**	7.33 ± 0.14	6.83 ± 0.63
**Bursa of fabricius**	7.33 ± 0.14	7.42 ± 0.14
**Thymus**	7.5	7.17 ± 0.38
**Pancreas**	4.83 ± 1.38	5.75 ± 0.5
**Cecal tonsils**	7.5 ± 0.25	6.67 ± 0.52

^a^Six-week-old chickens were inoculated intranasally (i.n.) with 10^6^ EID_50_ of each virus in a 0.2 ml volume; three chickens in each group were euthanized on 3 DPI, and virus titer was determined in samples of heart, liver, spleen, lung, kidney, brain, trachea, Small intestine, bursa of fabricius, Thymus, Pancreas and cecal tonsils in eggs [17].

^b^For statistical analysis, a value of 1.5 was assigned if the virus was not detected from the undiluted sample in three embryonated hen eggs. Virus titers are expressed as means ± standard deviation in log_10_EID_50_/g of tissue.

SS-10 and NH-10 viruses shedding from the inoculated chickens were detected in oropharyngeal and cloacal swabs at 3, 5, 7, 9 and 11 DPI. The SS-10 and NH-10 viruses could be tested from both oropharyngeal and cloacal swabs of inoculated chickens until they died within 6 DPI (Table 4). The SS-10 virus was shed from the oropharynx in inoculated chickens within 5 DPI, whose virus titers were from 2.67 log_10_EID_50_ to 4.30 log_10_EID_50_. It also could be shed from the cloaca within 5 DPI; the virus titers were from 1.75 log_10_EID_50_ to 4.5 log_10_EID_50_ (Table 4). NH-10 virus shedding could be tested from both oropharyngeal and cloacal swabs at 3 and 5 DPI. The virus titers were from 3.58 log_10_EID_50_ to 4.5 log_10_EID_50_ in oropharyngeal swabs and 4.04 log_10_EID_50_to 5.25 log_10_EID_50_ in cloacal swabs (Table 4).

**Table 4:** **Virus titers in cloacal and oropharyngeal swabs from chickens**

Strains		Days post-inoculation (log _10_EID _50_/0.1 mL) ± SD ^a^									
		3 Day		5 Day		7 Day		9 Day		11 Day	
		Oropharyngeal swabs	Cloacal swabs	Oropharyngeal swabs	Cloacal swabs	Oropharyngeal swabs	Cloacal swabs	Oropharyngeal swabs	Cloacal swabs	Oropharyngeal swabs	Cloacal swabs
**SS-10**	**Infected** ^**b**^	4.30 ± 0.51 (6/6)	4.5 (6/6)	2.67 ± 0.14 (3/3)	1.75 ± 0.43 (1/3)	-^d^	-	-	-	-	-
	**Contact** ^**c**^	2.50 ± 0.25 (3/3)	3.17 ± 0.63 (3/3)	3.42 ± 0.58 (3/3)	2.50 ± 0.75 (3/3)	1.75 (1/1)	1.75 (1/1)	ND^e^ (0/1)	ND (0/1)	ND (0/1)	ND (0/1)
**NH-10**	**Infected**	3.58 ± 0.20 (6/6)	4.04 ± 0.56 (6/6)	4.5 (1/1)	5.25 (1/1)	-	-	-	-	-	-
	**Contact**	2.42 ± 0.14 (3/3)	2.58 ± 0.14 (3/3)	4.25 ± 0.43 (3/3)	4.67 ± 0.88 (3/3)	-	-	-	-	-	-

^a^For statistical purposes, a value of 1.5 was assigned if virus was not detected from the undiluted sample in three embryonated hen’s eggs [17].

^b^Chickens inoculated with virus.

^c^Naive contact chickens housed with those inoculated.

^d^All the chickens died at the end of the observation.

^e^No detected.

To understand the horizontal transmission of these two viruses, three SPF chickens were inoculated intranasally with 0.2 ml phosphate buffered saline (PBS) as a naive contact group, which was housed with those inoculated with the SS-10 or NH-10 viruses.

During the observed time, two of the three naive contact chickens housed with SS-10 died on 6 DPI; the rest of the naive contact chickens seroconverted on 14 DPI (Table 4). The virus could be detected from oropharyngeal and cloacal swabs within 7 DPI. The virus titers were from 1.75 log_10_EID_50_ to 3.42 log_10_EID_50_ in oropharyngeal swabs and 1.75 log_10_EID_5_ to 3.17 log_10_EID_50_ in cloacal swabs (Table 4). All the chickens in the naive contact group, housed with inoculated SS-10 chickens, died within 5 DPI. The virus could be detected from oropharyngeal swabs and cloacal swabs at 3 and 5 DPI. The mean titers were from 2.42 log_10_EID_50_ to 4.25 log_10_EID_50_ in oropharyngeal swabs and 2.58 log_10_EID_5_ to 4.67 log_10_EID_50_ in cloacal swabs (Table 4).

Our results indicated that SS-10 and NH-10 viruses were highly pathogenic to chickens, and could transmit between chickens by naive contact.

**Pathogenicity and transmission in ducks** To determine the pathogenicity in ducks, we inoculated intranasally ducks with 10^6^ EID_50_ of SS-10 or NH-10 virus in a volume of 200 μl, and then observed the ducks for 2 weeks after infection.

No ducks died during the observation period. All the ducks seroconverted in the SS-10 and NH-10 groups (Table 2). The SS-10 virus caused systemic infection in ducks, including the spleen, lung, kidney, brain, trachea, small intestine, bursa of fabricius, thymus, pancreas and cecal tonsils. The virus replicated more highly in the thymus (4.08 log_10_EID_50_). The mean virus titers in the spleen, lung, kidney, brain, trachea, small intestine, bursa of fabricius, pancreas and cecal tonsils were 3.58, 2.58, 2.17, 1.58, 2.00, 2.75, 3.83, 2.17 and 2.67 log_10_EID_50_, respectively. The NH-10 virus replicated in some tested organs, including heart, lung, spleen, kidney, trachea, small intestine, bursa of fabricius, thymus, pancreas and cecal tonsils, but not the brain (Table 5). The mean virus titer was higher in the thymus (4.17 log_10_EID_50_).

**Table 5:** **Replication in ducks of NDV after inoculated intranasally** ^**a**^

Virus replication on 3 DPI(log _10_EID _50_/g) ^b^ in:	Strains	
	SS-10	NH-10
**Heart**	ND^**c**^	1.58 ± 0.14
**Liver**	ND	ND
**Spleen**	3.58 ± 0.14	3.58 ± 0.14
**Lung**	2.58 ± 0.14	2.50
**Kidney**	2.17 ± 0.95	2.08 ± 1.01
**Brain**	1.58 ± 0.14	ND
**Trachea**	2.00 ± 0.66	2.00 ± 0.66
**Small intestine**	2.75 ± 0.43	3.42 ± 0.88
**Bursa of fabricius**	3.83 ± 0.38	3.50
**Thymus**	4.08 ± 1.01	4.17 ± 0.95
**Pancreas**	2.17 ± 0.95	1.67 ± 0.14
**Cecal tonsils**	2.67 ± 0.14	2.58 ± 0.14

^a^Two-week-old ducks were inoculated intranasally (i.n.) with 10^6^ EID_50_ of each virus in a 0.2 ml volume; three ducks in each group were euthanized on 3 DPI, and virus titer was determined in samples of heart, liver, spleen, lung, kidney, brain, trachea, Small intestine, bursa of fabricius, Thymus, Pancreas and cecal tonsils in eggs [17].

^b^For statistical analysis, a value of 1.5 was assigned if the virus was not detected from the undiluted sample in three embryonated hen eggs. Virus titers are expressed as means ± standard deviation in log_10_EID_50_/g of tissue.

^c^No detected.

SS-10 and NH-10 viruses shedding from the inoculated ducks were detected in oropharyngeal and cloacal swabs at 3, 5, 7, 9 and 11 DPI. The SS-10 virus could be detected in both oropharyngeal and cloacal swabs within 3 DPI (Table 6). The mean virus titers in the oropharyngeal and cloacal swabs were 1.96 log_10_EID_50_ and 1.89 log_10_EID_50_, respectively. In the NH-10 group, the virus titers of oropharyngeal swabs could only be detected at 3 DPI (2.17 log_10_EID_50_). The virus titers of the cloacal swabs were from 1.58 log_10_EID_50_ to 2.17 log_10_EID_50_ within 7 DPI.

**Table 6:** **Virus titers in cloacal and oropharyngeal swabs from ducks**

Strains		Days post-inoculation (log _10_EID _50_/0.1 mL) ± SD ^a^									
		3 Day		5 Day		7 Day		9 Day		11 Day	
		Oropharyngeal swabs	Cloacal swabs	Oropharyngeal swabs	Cloacal swabs	Oropharyngeal swabs	Cloacal swabs	Oropharyngeal swabs	Cloacal swabs	Oropharyngeal swabs	Cloacal swabs
**SS-10**	**Infected** ^**b**^	1.96 ± 0.60(4/6)	1.89 ± 0.76(2/6)	ND^d^ (0/3)	ND (0/3)	ND (0/3)	ND (0/3)	ND (0/3)	ND (0/3)	ND (0/3)	ND (0/3)
	**Contact** ^**c**^	1.75 ± 0.43(1//3)	ND (0/3)	2.25 ± 1.30(1/3)	2.00 ± 0.43(2/3)	ND (0/3)	1.83 ± 0.57(1/3)	ND (0/3)	ND (0/3)	ND (0/3)	ND (0/3)
**NH-10**	**Infected**	2.17 ± 1.04 (2/6)	2.17 ± 1.04 (2/6)	ND (0/3)	1.58 ± 0.14(1/3)	ND (0/3)	1.58 ± 0.14(1/3)	ND(0/3)	ND(0/3)	ND(0/3)	ND(0/3)
	**Contact**	1.58 ± 0.14(1/3)	ND (0/3)	ND (0/3)	ND (0/3)	ND(0/3)	ND(0/3)	ND(0/3)	ND(0/3)	ND(0/3)	ND(0/3)

^a^For statistical purposes, a value of 1.5 was assigned if virus was not detected from the undiluted sample in three embryonated hen’s eggs [17].

^b^Ducks inoculated with virus.

^c^Naive contact ducks housed with those inoculated.

^d^No detected.

To study the horizontal transmission of these two viruses, three ducks were inoculated intranasally with 0.2 ml phosphate buffered saline (PBS) as a naive contact group housed with those inoculated with the viruses previously mentioned.

The naive contact ducks, housed with the ducks inoculated SS-10 or NH-10, experienced no deaths during the test time. SS-10 viruses shedding of the naive contact group could be detected in the oropharyngeal and the cloacal swabs. The virus titers were from 1.75 log_10_EID_50_ to 2.25 log_10_EID_50_ within 5 DPI in oropharyngeal swabs and 1.83 log_10_EID_5_ to 2.00 log_10_EID_50_ within 7 DPI in cloacal swabs, respectively (Table 6). In the naive contact group of NH-10, The virus could be detected from oropharyngeal swabs at 3 DPI(1.58 log_10_EID_50_). According to these, the shedding time and virus titers of SS-10 virus were longest and highest among the two viruses.

In the ducks study, these results indicate that SS-10 and NH-10 viruses could infect ducks and transmit between ducks by naive contact.

**Pathogenicity and transmission in geese** To evaluate the pathogenicity of the two viruses in geese, we inoculated intranasally with 10^6^ EID_50_ of each virus in a volume of 200 μl in geese, and then observed them for 14 days after infection.

In the inoculated group, SS-10 caused one of the three inoculated geese to die on 6 DPI, but no geese from the NH-10 groups died during the 14-day observation period. All the surviving geese seroconverted in the SS-10 and NH-10 groups (Table 2). The SS-10 and NH-10 viruses replicated systemically in geese, which could be detected from all the tested organs except brain and heart on 3 DPI, including the heart, liver, spleen, lung, kidney, brain, trachea, small intestine, bursa of fabricius, thymus, pancreas and cecal tonsils. The SS-10 or NH-10 viruses replicated highly in spleen; the mean titers were 5.58 log_10_EID_50_ and 5.75 log_10_EID_50_, respectively (Table 7).

**Table 7:** **Replication in geese of the NDV viruses after inoculated intranasally** ^**a**^

Virus replication on 3 DPI(log _10_EID _50_/g) ^b^ in:	Strains	
	SS-10	NH-10
**Heart**	ND^**c**^	ND
**Liver**	2.08 ± 1.01	2.17 ± 0.95
**Spleen**	5.58 ± 1.01	5.75 ± 0.50
**Lung**	3.83 ± 0.38	3.08 ± 0.58
**Kidney**	3.5 ± 1.75	3.00 ± 1.39
**Brain**	ND	ND
**Trachea**	1.92 ± 0.72	1.92 ± 0.72
**Small intestine**	4.08 ± 0.58	3.00 ± 1.39
**Bursa of fabricius**	4.00 ± 0.66	4.50 ± 0.75
**Thymus**	4.92 ± 0.52	5.33 ± 0.52
**Pancreas**	2.58 ± 1.01	1.92 ± 0.28
**Cecal tonsils**	4.08 ± 0.58	4.83 ± 0.38

^a^Two-week-old geese were inoculated intranasally (i.n.) with 10^6^ EID_50_ of each virus in a 0.2 ml volume; three ducks in each group were euthanized on 3 DPI, and virus titer was determined in samples of heart, liver, spleen, lung, kidney, brain, trachea, Small intestine, bursa of fabricius, Thymus, Pancreas and cecal tonsils in eggs [17].

^b^For statistical analysis, a value of 1.5 was assigned if the virus was not detected from the undiluted sample in three embryonated hen eggs. Virus titers are expressed as means ± standard deviation in log_10_EID_50_/g of tissue.

^c^No detected.

SS-10 and NH-10 viruses shedding from the inoculated ducks were detected in oropharyngeal and cloacal swabs at 3, 5, 7, 9 and 11 DPI. In the SS-10 group, the virus titers of the oropharyngeal swabs were from 1.67 log_10_EID_50_ to 2.67 log_10_EID_50_ within 5 DPI. The virus titers of cloacal swabs were from 1.60 log_10_EID_50_ to 1.92 log_10_EID_50_ within 7 DPI (Table 8). The NH-10 virus was shed from the oropharynx in inoculated geese within 7 DPI, whose virus titers were from 1.58 log_10_EID_50_ to 2.38 log_10_EID_50_. It also could be shed from the cloaca within 5 DPI; the virus titers were from 1.75 log_10_EID_50_ to 2.08 log_10_EID_50_ (Table 8).

**Table 8:** **Virus titers in cloacal and oropharyngeal swabs from geese**

Strains		Days post-inoculation (log _10_EID _50_/0.1 mL) ± SD ^a^									
		3 Day		5 Day		7 Day		9 Day		11 Day	
		Oropharyngeal swabs	Cloacal swabs	Oropharyngeal swabs	Cloacal swabs	Oropharyngeal swabs	Cloacal swabs	Oropharyngeal swabs	Cloacal swabs	Oropharyngeal swabs	Cloacal swabs
**SS-10**	**Infected** ^**b**^	2.67 ± 0.79(5/6)	1.60 ± 0.14(3/6)	1.67 ± 0.14(2/3)	1.92 ± 0.72(1/3)	ND ^d^ (0/3)	1.88 ± 0.53(2/3)	ND(0/3)	ND(0/3)	ND(0/3)	ND(0/3)
	**Contact** ^**c**^	1.58 ± 0.14(1/3)	2.08 ± 1.01(2/3)	1.58 ± 0.14 (1/3)	3.00 ± 1.30(2/3)	ND(0/3)	ND(0/3)	ND(0/3)	ND(0/3)	ND(0/3)	ND(0/3)
**NH-10**	**Infected**	2.38 ± 0.38(6/6)	2.08 ± 0.90(2/6)	2.17 ± 1,15(1/3)	1.75 ± 0.43(1/3)	1.58 ± 0.14(1/3)	ND(0/3)	ND(0/3)	ND(0/3)	ND(0/3)	ND(0/3)
	**Contact**	3.17 ± 0.95(3/3)	2.00 ± 0.66(2/3)	1.58 ± 0.14(1/3)	1.75 ± 0.43(1/3)	1.58 ± 0.14(1/3)	1.58 ± 0.14(1/3)	ND(0/3)	ND(0/3)	ND(0/3)	ND(0/3)

^a^For statistical purposes, a value of 1.5 was assigned if virus was not detected from the undiluted sample in three embryonated hen’s eggs [17].

^b^Geese inoculated with virus.

^c^Naive contact geese housed with those inoculated.

^d^No detected.

To understand the horizontal transmission of these two viruses, three geese were inoculated intranasally with 0.2 ml phosphate buffered saline (PBS) as a naive contact group, which was housed with those inoculated with the two viruses described previously.

The SS-10 virus caused one of the three naive contact geese to die, and could be isolated from oropharyngeal and cloacal swabs during the observed period. The mean virus titers of the oropharyngeal swabs were 1.58 log_10_EID_50_ within 5 DPI (Table 8). And the virus titers of cloacal swabs could be detected from 2.08 log_10_EID_50_ to 3.00 log_10_EID_50_ at 3 and 5 DPI (Table 8). In the naive contact group of NH-10, the virus titers of the oropharyngeal swabs were from 1.58 log_10_EID_50_ to 3.17 log_10_EID_50_ within 7 DPI. The virus could be detected from oropharyngeal and cloacal swabs within 7 DPI. The virus titers were from 1.58 log_10_EID_50_ to 3.17 log_10_EID_50_ in oropharyngeal swabs and 1.58log_10_EID_5_ to 2.00 log_10_EID_50_ in cloacal swabs (Table 8).

Our results indicated that SS-10 virus was highly pathogenic to geese, and could transmit between geese by naive contact. The NH-10 virus could infect geese and transmit between geese by naive contact.

## Discussion

Historically, ND has been a devastating disease in poultry, and in many countries the disease remains one of the major problems affecting existing and developing poultry industries. Even in countries where ND may be considered to be controlled, an economic burden is still associated with vaccination and maintaining strict biosecurity measures [2]. Around the world, over 241 different bird species have been reported to be susceptible to NDV as a result of natural or experimental infections, and it is likely that many more susceptible species exist but have not yet been identified.

Though waterfowl are considered the natural reservoirs of NDVs and mostly harbor lentogenic strains, surveillance for avirulent Newcastle disease viruses in domestic ducks were regularity isolated from duck farms in the China [14, 18]. In this study, two strains isolated from China were genotypically and pathotypically characterized during 2010. Pathogenicity results revealed that both isolates (SS-10 and NH-10) were velogenic. The two NDV isolates isolated from ducks were highly pathogenic for chickens and geese, but low pathogenic for ducks. This suggests that NDV from waterfowl were highly pathogenic to terrestrial birds and waterfowl, and might transmit among them. The two NDV exhibited different biological properties with respect to pathogenicity and transmission in chickens, ducks and geese. The SS-10 virus isolated from ducks was highly pathogenic for chickens and geese, but low pathogenic for ducks. Moreover, it could transmit to chickens, ducks and geese by naive contact. However, the NH-10 virus isolated from ducks could infect some chickens, ducks and geese, but only caused chickens to die. Additionally, it could transmit to the naive contact chickens, ducks, and geese. Therefore, our findings showed that the pathogenicity and transmission in birds of the two viruses varied. This suggests that the presence of multiple NDV strains in South China and the highly transmissible nature of the virus can complicate and increase the cost of attempts to prevent the spread of infection to the rest of china and other parts of the world.

Even though most of duck-origin NDV strains are non-pathogenic in ducks, duck-origin NDV strains remain potential threats to the poultry industry. Firstly, the two viruses are able to replicate in most tissues in ducks, especially in the lymphoid tissues (spleen, bursa of fabricius, thymus and cecal tonsils), even though there were no clinical signs or gross lesions, indicating that ducks may carry duck-origin NDV strains without overtsigns of clinical disease and are likely to shed viruses via the oral or cloacal route. Secondly, duck-origin NDV strains passaged in ducks enhanced virulence (date not shown). Finally, the two NDV viruses exhibited highly pathogenic for chickens and geese, and replicated systemically in the inoculated chickens and geese. Therefore, outbreaks by highly pathogenic NDVs can occur by the introduction of benign viruses into waterfowl followed by serial passages under natural conditions, emphasizing the importance of the host environment in the process of NDV selection.

Nowadays, vaccine strains Lasota, B1 and V4 are used widely in China to control the infection of NDV. However, virulent NDV strains are still isolated frequently in the vaccinated birds [14], which demonstrates that NDV remains an on-going threat to commercial flocks. The outbreaks of Newcastle disease in the vaccinated poultry flocks raised concerns on the protective efficiency of commercially available vaccines. Sporadic cases of ND in commercial farms vaccinated with Lasota vaccine were previously reported in China [11, 19, 20], in Southern California and adjacent states [21], in Indonesia [22], Venezuela [23], Egypt [24], Nigeria and Burkina Faso [25]. It is however possible that NDV strains responsible for ND sporadic outbreaks in vaccinated chickens can escape the immune responses, and thus contribute to the emergence of new genotypes [10]. Thus, the control of NDV by vaccination still faces new challenges in different avian species.

To sum up, it is an urgent to take stricter biosecurity measures in poultry breeding management, in particular to avoid mixing different types of poultry rearing in order to reduce cross-species transmission. Additionally, we also strengthen immunoprophylaxis and regular monitoring of ducks, geese and other waterfowl of the NDV geographic distribution. The present investigation provides important information on the epidemiology, diagnosis and control of NDV in South China and highlights the importance of supporting surveillance in developing countries for transboundary animal diseases.

## Materials and methods

### Viruses

In 2010, two Newcastle disease viruses Duck/CH/GD/SS/10 (SS-10) and Duck/CH/GD/NH/10 (NH-10) were isolated from cloacal swabs of apparently healthy birds in live bird markets during 2010. All viruses were propagated in 10-day-old embryonated specific-pathogen-free (SPF) chicken eggs. All experiments were carried out in animal biosafety level 3 (ABSL-3) facilities. All animal procedures performed in this study were reviewed, approved, and supervised by the Guangdong Administration Committee of Laboratory Animals under the leadership of the Guangdong Association for Science and Technology, permit number SCXK(Guangdong)2008-0019.

### Assessment of the biological characteristics of two isolates

The pathogenic potential for the two isolated viruses were evaluated using standard assay methods to determine the MDT in 10-day-old chick embryos and the ICPI in 1-day-old chicks [2]. The 50% embryo infectious doses (EID_50_) of two isolates were also determined with 10-day-old chick embryos and calculated by the method of Reed and Muench [26].

### Complete genome amplification of these two virus strains by RT-PCR

Viral RNA was isolated from these two NDV-infected allantoic fluids of 10-day-old SPF chicken embryos, using an RNeasy mini kit (Qiagen, Valencia, CA) according to manufacturer’s recommendations and transcribed into cDNA with SuperScript III reverse transcriptase (Invitrogen). Amplification reactions were performed with the one-step RT-PCR kit (Qiagen, Valencia, CA). PCR was performed using fragment-specific primers. A set of 12 pairs of specific primers (see Table 9) were designed to amplify the complete genome of SS-10 and NH-10 which were based on the available NDV nucleotide sequences (GM, ZJ1, JS/1/02/Du, F48E9, JS/1/97/Ch, ZJ/1/86/Ch, and Duck/1/05, with GenBank accession numbers DQ486859, AF431744, FJ436306, AY508514, FJ436305, FJ436303 and EU649675, respectively). The PCR products of the expected length were subjected to electrophoresis in 1% agarose gels purified by using the QuickClean DNA gel extraction kit (Qiagen, Valencia, CA).

**Table 9:** **Primers used in the study**

Primers	Sequences (5′-3′)	Position	Expected extent (bp)	Expected size (bp)
1F^a^	ACCAAACAGAGAATCCGTGAG	1-21	1-1 270	1 270
1R^b^	CTTTAGCTCGGCAGCCATATCCTC	1258-1270		
2F	TGAGCACATCATTCTGGAGACTTG	1185-1207	1 185–2 280	1 096
2R	TGGACGATTTATTGCTAAGCTTG	2259-2280		
3F	CTAAGCACAGCATGGGAGAAG	2025-2046	2 025–3 432	1 408
3R	AAGTCAAGACGCTGGATCCT	3413-3432		
4F	CACAGGAGATGGGAAGAAGC	3376-3395	3 376–5 330	1 955
4R	ATGAGCTGAGTTGATTGTTCC	5310-5330		
5F	CAGTTGGGAAGATGCAGCAG	5076-5095	5 076–6 873	1 798
5R	AGAGGGATAGAATGATGTGAC	6853-6873		
6F	CCTGTTCATGACCCAGACTAC	6787-6807	6 787–8 520	1 734
6R	TCGAAGTCACATTCATCAGG	8501-8520		
7F	GATCAGATGAGAGCCACTACAA	6471-6492	6 471–8 448	1 978
7R	GATAGATGTGACTCTGGTAGGAT	8426-8448		
8F	AGAGGGAACACGGGTAGGA	8376-8394	8 367–10 054	1 688
8R	CTTAGCAAAAATCCGCCCA	10036-10054		
9F	ATCGTACTCACTCAAAGAGA	10000-10019	10 000–11 747	1 748
9R	GGGCATAATCTGCTAGCGTC	11728-11747		
10F	TGACGCTAGCAGATTATG	11727-11744	11 727–13 422	1 696
10R	TCCTGAGGGAACAACAAT	13405-13422		
11F	TCTTTCCAATGACAACAACC	12604-12623	12 604–14 589	1 986
11R	GACCGGATAACTGTGTCAAT	14570-14589		
12F	AAGATTCGTCCAGGGTCC	14033-14050	14 033–15 192	1 160
12R	ACCAAACAGAGATTTGGTGAA	15172-15192		

^a^F stands for forward primer.

^b^R stands for reverse primer.

### Nucleotide sequencing and phylogenetic analysis

The purified PCR products were sequenced using an automatic ABI Prism 3730 genetic analyzer (Applied Biosystems) according to the manufacturer’s instructions. Nucleotide sequence editing, analysis, the prediction of amino acid sequences and alignments were performed using the MegAlign program in the Lasergene package (Laser-Gene, version 5.07; DNAStar, Inc., Madison, WI). The sequences of the two isolates were compared with 62 previously reported NDV sequences representative of different genotypes available in GenBank (Additional file 1: Table S1). Phylogenetic trees were constructed by the neighbor-joining method of MEGA 4 [16] by a comparison of the nucleotide sequences of the entire ORF of the F gene from 1 to 1,662 nucleotides (nt) and the complete genome from 1 to 15192 nt [27]. The reliability of the tree was assessed by bootstrap analysis with 1000 replicates. Horizontal distances are proportional to genetic distance. Putative recombination events in the two full-genomes of SS-10 and NH-10 isolates were examined using the SimPlot program (version 3.5) [28].

### Animal experiment

#### Pathogenicity and transmission in chickens

Six-week-old SPF White Leghorn chickens were purchased from Guangdong Dahuanong and housed in the isolator cages and randomly divided into 2 groups. Six chickens of the SS-10 group were inoculated intranasally with 10^6^ EID_50_ of SS-10 viruses in a 0.2 ml volume. Six chickens of the NH-10 group were inoculated intranasally with 10^6^ EID_50_ of NH-10 viruses in a 0.2 ml volume Three chickens were inoculated intranasally with 0.2 ml phosphate buffered saline (PBS) as naïve contact group housed with those inoculated with the SS-10 or NH-10 viruses, respectively. All chickens were observed for clinical symptoms for 14 days. On 3-day post-inoculated (DPI), three inoculated chickens in each group were euthanized to test the virus replication in organs, including heart, liver, spleen, lung, kidney, brain, trachea, small intestine, bursa of fabricius, thymus, pancreas and cecal tonsils. Similar actions were performed on chickens that had died. Oropharyngeal and cloacal swabs were taken from chickens at 3, 5, 7, 9 and 11 DPI, and suspended in 1 ml PBS. All of the tissues and swabs were collected and titrated for virus infectivity in SPF chick embryos. Seroconversion of the surviving birds on 14 DPI was confirmed by hemagglutinin inhibition (HI) test.

#### Pathogenicity and transmission in ducks

Eighteen two-week-old healthy Peking ducks were purchased from a duck farm in Guangzhou and housed in the isolators and randomly divided into 2 groups. Peking ducks were confirmed serologically negative for Newcastle disease by hemagglutination inhibition (HI) assays. Six of nine ducks were inoculated intranasally with 10^6^ EID_50_ of each virus in a 0.2 ml volume. The other three ducks were inoculated intranasally with 0.2 ml PBS as a naive contact group housed with those inoculated with the SS-10 or NH-10 viruses, respectively. Three inoculated ducks in each group were euthanized to test the virus replication in organs on 3 DPI, including heart, liver, spleen, lung, kidney, brain, trachea, small intestine, bursa of fabricius, thymus, pancreas and cecal tonsils, and the six remaining ducks were observed for clinical symptoms (inappetence, lethargy, greenish or white watery diarrhoea, dyspnoea, tremors, morbidity and mortality) for 14 days. Swabs from the oropharynx and cloaca were collected for detection of viruses shedding at 3, 5, 7, 9 and 11 DPI. All of the tissues and swabs were collected and titrated for virus infectivity in SPF chick embryos. Seroconversion of the surviving birds on 14 DPI was confirmed by HI test.

#### Pathogenicity and transmission in geese

Two groups of two-week-old healthy domestic geese (Qingyuan geese) were purchased from a goose farm in Guangzhou and housed in isolators. Geese were confirmed serologically negative for Newcastle disease by hemagglutination inhibition (HI) assays. Six geese of each group were inoculated intranasally with 10^6^ EID_50_ of each virus in a 0.2 ml volume. Three geese were inoculated with 0.2 ml PBS as a naive contact group housed with those inoculated with the SS-10 or NH-10 viruses, respectively. On 3 DPI, three inoculated geese in each group were euthanized to test the virus replication in organs, including heart, liver, spleen, lung, kidney, brain, trachea, small intestine, bursa of fabricius, thymus, pancreas and cecal tonsils. Oropharyngeal and cloacal swab specimens were collected at 3, 5, 7, 9 and 11 DPI. All of the tissues and swabs were collected and titrated for virus infectivity in SPF chick embryos. Seroconversion of the surviving birds on 14 DPI was confirmed by HI test.

## Electronic supplementary material

Additional file 1: Table S1: NDV isolates and their accession numbers used in phylogenetic analysis. (DOC 93 KB)
