# Supplementary material for: Phylogenetic relationships and pathogenicity variation of two Newcastle disease viruses isolated from domestic ducks in Southern China
Source: Virol J. 2014 Aug 12;11:147. doi: 10.1186/1743-422X-11-147 (PMC4254411; doi:10.1186/1743-422X-11-147)
Supplement: Supplementary file 1 — Additional file 1: Table S1: NDV isolates and their accession numbers used in phylogenetic analysis. (DOC 93 KB) [file 12985_2014_2475_MOESM1_ESM.doc]

| Accession number | Virus strains | Year | Country | Genotype or Class |
| --- | --- | --- | --- | --- |
| DQ486859 | GM |  | China | VIId |
| AY562985 | Cockatoo/Indonesia/14698/90 | 1990 | Indonesia | VIIa |
| AF217084 | Queensland/V4 |  | Australia | I |
| M24698 | Texas GB/48 | 1948 | USA | II |
| X04719 | Beaudette C/ | 1945 | USA | II |
| NC_002617 | B1 | 1947 | USA | II |
| Y18898 | Clone/30 |  |  | II |
| AY845400 | LaSota | 1946 | USA | II |
| AY225110 | HB92 |  | China | II |
| M18456 | JP/Miyadera/51 | 1951 | Japan | III |
| M21881 | AUS/Victoria/32 | 1932 | Australia | III |
| EF201805 | Mukteswar |  | China | III |
| AY741404 | Herts/33 |  | Netherands | IV |
| EU293914 | Italien |  | China | IV |
| AY288999 | Chicken/Mexico/37821/96 | 1996 | Mexico | V |
| AY562987 | Gamefowl/U.S(CA)/211472/02 | 2002 | USA | V |
| AY562986 | Anhinga/U.S(FL)/44083/93 | 1993 | USA | V |
| AY562989 | Dove/Italy/2736/00 | 2000 | Italy | VI |
| AJ880277 | IT-227/82 |  | Italy | VI |
| AY865652 | Sterna/Astr/2755/2001 | 2001 | Australia | VIIb |
| AF431744 | ZJI | 2000 | China | VIId |
| DQ659677 | NA/1 | 2006 | China | VIId |
| AF358786 | TW/2000 | 2000 | Taiwan | VIId |
| AF456442 | JS/5/01 /Go | 2001 | China | VIId |
| FJ872531 | Muscovy duck/China(Fujian)/FP1/02 | 2009 | China | VII |
| GQ849007 | JSD0812 | 2009 | China | VII |
| EF589132 | DQ |  | China | VII |
| DQ682437 | JS/1/03/Go | 2008 | China | VII |
| HM063425 | P4 | 2010 | China | VI |
| AY508514/ AY997298 | F48E9 | 1948 | China | IX |
| FJ480789 | CK/CH/GD/1/05 | 2008 | China | VII |
| FJ436306 | JS/1/02/Du | 2009 | China | IX |
| GQ853450 | ND-XX08 | 2009 | China | VII |
| DQ682448 | JS/1/04/Go | 2008 | China | VII |
| DQ682450 | SD/1/04/Go | 2008 | China | VII |
| FJ436305 | JS/1/97/Ch | 2009 | China | IX |
| DQ469830 | JS/1/05 | 2006 | China | VII |
| DQ234584 | SRZ03 | 2005 | China | VII |
| EU649675 | Duck/1/05 | 2008 | China | VII |
| FJ436303 | ZJ/1/86/Ch | 2009 | China | IX |
| FJ492892 | Duck/China/SD23/2008 | 2010 | China | Class I |
| FJ492893 | Duck/China/SD27/2008 | 2010 | China | Class I |
| AY562991 | isolate chicken/N. Ireland/Ulster/67 |  | Ireland | I |
| KC542914 | Chicken/China/Hebei/01/2012 | 2012 | China | VII |
| KC542894 | Chicken/China/Zhejiang/01/2006 | 2006 | China | VII |
| JX244791 | Pigeon/China/SD/54/06 | 2006 | China | VII |
| JN631747 | JS/5/05/Go | 2005 | China | VII |
| GQ245798 | CZ/10/08/Ch | 2008 | China | VII |
| GQ245808 | XY/31/07/Ch | 2007 | China | VII |
| FJ480789 | CK/CH/GD/1/05 | 2005 | China | VII |
| DQ485274 | dove/Guangxi/15/2005 | 2005 | China | VII |
| GU124591 | sh09 | 2005 | China | VII |
| AY028995 | Ch/A7/96 | 2001 | China | VIIc |
| AY288996 | pigeon/Italy/1166/00 | 2004 | USA | VI |
| FJ751919 | QH4 | 1985 | China | VIII |
| HQ266603 | MG_1992 | 1992 | Madagascar | XI |
| FJ436304 | FJ/1/85/Ch | 2011 | China | IX |
| FJ608361 | GM/Shandong/01 | 2001 | China | VII |
| HM063423 | W4 | 2005 | China | VI |
| AY289001 | turkey/USA(ND)/43084/92 | 2004 | USA | V |
| FJ600541 | FJ0801 | 2001 | China | I |
| EF612277 | Northern Pintail/US(AK)/196/1998 | 1998 | USA | class I |
